# Wikipedia page views for health research: a review

**DOI:** 10.3389/fdata.2023.1199060

**Published:** 2023-07-04

**Authors:** Rowalt Alibudbud

**Affiliations:** Department of Sociology and Behavioral Sciences, De La Salle University, Manila, Philippines

**Keywords:** Wikipedia, health research, research methodology, health informatics, internet based intervention, infodemiology, online information

## Abstract

Wikipedia is an open-source online encyclopedia and one of the most-read sources of online health information. Likewise, Wikipedia page views have also been analyzed to inform public health services and policies. The present review analyzed 29 studies utilizing Wikipedia page views for health research. Most reviewed studies were published in recent years and emanated from high-income countries. Together with Wikipedia page views, most studies also used data from other internet sources, such as Google, Twitter, YouTube, and Reddit. The reviewed studies also explored various non-communicable diseases, infectious diseases, and health interventions to describe changes in the utilization of online health information from Wikipedia, to examine the effect of public events on public interest and information usage about health-related Wikipedia pages, to estimate and predict the incidence and prevalence of diseases, to predict data from other internet data sources, to evaluate the effectiveness of health education activities, and to explore the evolution of a health topic. Given some of the limitations in replicating some of the reviewed studies, future research can specify the specific Wikipedia page or pages analyzed, the language of the Wikipedia pages examined, dates of data collection, dates explored, type of data, and whether page views were limited to Internet users and whether web crawlers and redirects to the Wikipedia page were included. Future research can also explore public interest in other commonly read health topics available in Wikipedia, develop Wikipedia-based models that can be used to predict disease incidence and improve Wikipedia-based health education activities.

## 1. Introduction

Wikipedia is an open-source online encyclopedia available in over 275 languages and has more than 32 million articles across various topics, including health and medicine (Heilman and West, 2015). Since its inception in 2001, it has been an influential public health platform and one of the most commonly read sources of online health information (Shafee et al., 2017). However, despite its popularity and broad content, Wikipedia faces many challenges, including its small and decreasing core editors and the academic world's skepticism (Heilman and West, 2015; Jemielniak, 2019).

Despite criticisms and skepticism from academics, a review of health-related articles on Wikipedia revealed that they commonly referenced several respected journals, such as The Lancet, The New England Journal of Medicine, Nature, Journal of the American Medical Association, and Science, were the most commonly cited sources (Heilman and West, 2015). Additionally, approximately half of its core editors are healthcare providers (Heilman and West, 2015). Furthermore, Wikipedia content often attains high rankings in Google search results (Smith, 2020; Mendes et al., 2021). Further examination of web traffic patterns has indicated that Wikipedia surpasses institutional health websites in terms of its online presence. Notably, it garners higher web traffic compared to esteemed platforms such as the National Institutes of Health, WebMD, Mayo Clinic, National Health Service, and the World Health Organization (Heilman and West, 2015). Likewise, a scoping review reveals that 50–70% of physicians and over 90% of medical students used it as a source of health information (Shafee et al., 2017). Thus, there is high utilization of Wikipedia for health information despite criticisms. Consequently, Wikipedia page views, similar to other big data from the Internet (e.g., Google search volume), have been analyzed to inform public health services and policies (Alibudbud and Cleofas, 2022; Alibudbud, 2023b).

Eysenbach (2009) defined infodemiology as “the science of distribution and determinants of information in an electronic medium, specifically the Internet, or in a population, with the ultimate aim to inform public health and public policy.” Therefore, using Wikipedia page views, which are information from the Internet, to inform public health services and policies can be subsumed under infodemiology.

### 1.1. Objectives and significance

This review explored the use of Wikipedia page views for health research. Publications were summarized and described according to their year of publication, authors' country of origin, health topic, purpose, data analysis, and other utilized big data from the Internet. By doing so, it summarizes the current state of the art and informs future research of the extent and considerations in using Wikipedia page views data.

## 2. Methods

This review included studies utilizing Wikipedia page views for health research from PubMed, one of the world's largest health research databases, and Scopus, one of the world's largest research databases. Specifically, research publications in the English language utilizing Wikipedia page views for health-related topics until March 2023 were included in this review. Letters, abstracts, those primarily about non-health-related topics (e.g., conservation), and those not written in English were excluded. The keyword used to search for relevant publications were “Wikipedia” and “page” and “views”.

**Figure 1:**
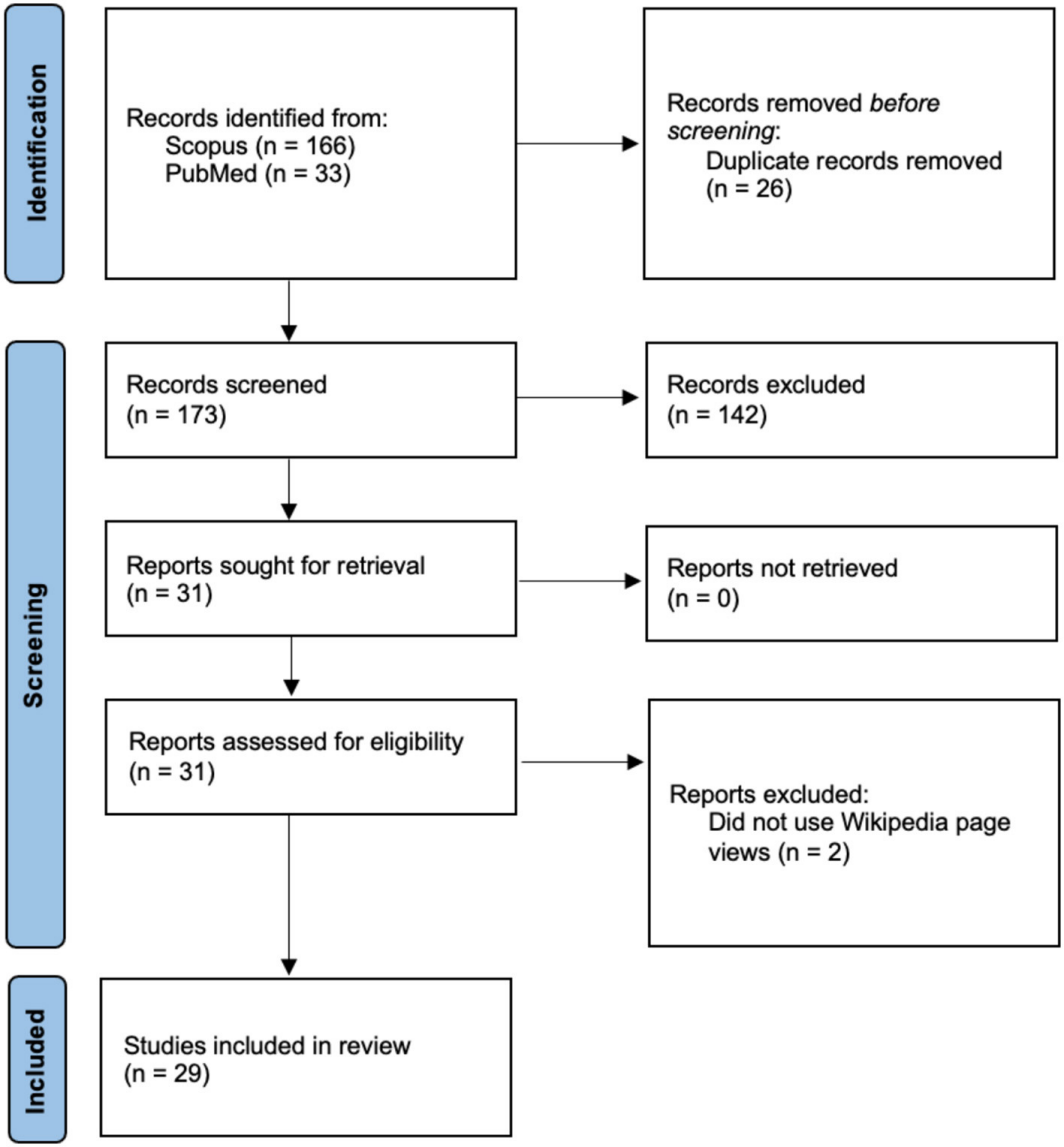
Flow diagram of the review.

Figure 1 shows that 166 and 33 publications were collected from Scopus and PubMed after searching for titles, abstracts, and keywords. After collecting the articles from each database, 26 duplicate articles were removed. Then, each publication was screened for eligibility based using its abstract and title, including being written in English, the use of Wikipedia page views, and topics related to health. After excluding 142 during the eligibility screening, studies were sought and mainly assessed based on their year of publication, authors' country of origin, health topic, purpose, data analysis, and whether they utilized other sources of internet big data. Upon further assessment, two additional publications were removed since they did not use Wikipedia page views. Thus, a total of 29 studies were included in this review.

## 3. Results and discussion

### 3.1. Publication years and countries of origin

The most productive year was 2021 (see Supplementary material), with eight publications (*n* = 8, 27.595), followed by 2020 (*n* = 5, 17.24%) and 2022 (*n* = 4, 13.79%). The most productive country was Italy, with 12 publications (41.38%), followed by the United States (*n* = 10, 34.48%) and the United Kingdom (*n* = 4, 13.79%). Thus, most of the reviewed studies have been published in recent years. Likewise, a disparity between high-income and low- and middle-income countries was found, where most of the reviewed studies emanated from high-income countries (e.g., Italy and the United States) than low- and middle-income countries (LMICs) (e.g., Philippines and Nigeria). Therefore, future research using Wikipedia page views can be undertaken in LMICs, especially about diseases more prevalent in these countries than in high-income nations (e.g., Tuberculosis in the Philippines).

### 3.2. Other utilized internet data

Wikipedia page views were most commonly combined with Google data (e.g., Google Trends, Google Analytics) (*n* = 16, 55.17%), followed by Twitter data (e.g., Twitter mentions) (*n* = 5, 17.24%), and Pubmed and Medline (*n* = 4, 13.79%). The reviewed studies also combined Wikipedia page views with other internet data, including online news, Youtube, Reddit, and Wikipedia Edit data (Sciascia and Radin, 2017; Gozzi et al., 2020; Szmuda et al., 2020; Wang and Zhang, 2020).

In general, while Wikipedia is one of the most highly utilized sources of health information, internet users may also explore other health websites for their needed information (Heilman and West, 2015). To address this limitation, most reviewed studies utilized these other internet data sources to expand their coverage and understand the patterns of online information utilization.

### 3.3. Health topics explored using Wikipedia page views

Wikipedia page views have also been used to understand various health topics. The most common topic explored using Wikipedia page views by the reviewed studies were non-communicable diseases (*n* = 11, 37.93%), followed by communicable diseases (*n* = 7, 24.14%), factors related to health (*n* = 2, 6.90%), medications (*n* = 2, 6.90%), and a combination of the aforementioned topics (*n* = 6, 20.69%). Two (6.90%) of the reviewed studies did not indicate the specific Wikipedia pages they explored.

The specific topics explored by the reviewed studies included Dementia (Brigo et al., 2015; Alibudbud, 2023b), fencing response (Roe et al., 2023), tumors (e.g., pancreatic tumors, brain tumors, colorectal cancer) (Naik et al., 2021; Mondia et al., 2022; Gianfredi et al., 2023), substance use disorder (Alibudbud and Cleofas, 2022), epilepsy (Brigo et al., 2015; Okumura et al., 2016), schizophrenia (Adams et al., 2020), diabetes mellitus (Potapov et al., 2021), pain (e.g., migraine, low back pain, inflammation, sciatica) (Brigo et al., 2015; Szmuda et al., 2020; Ciaffi et al., 2021; Potapov et al., 2021), cardiovascular diseases (Potapov et al., 2021), gastrointestinal conditions (Potapov et al., 2021), dermatological agents (Potapov et al., 2021), viral infections (e.g., coronavirus, COVID-19, influenza, Chikungunya) (Laurent and Vickers, 2009; Mahroum et al., 2018; Provenzano et al., 2019; Qiu et al., 2019; Gozzi et al., 2020; O'Leary and Storey, 2020; De Toni et al., 2021; Gianfredi et al., 2021; Rutovic et al., 2021; Storey and O'Leary, 2022), autoimmune conditions (e.g., Systemic Lupus Erythematosus) (Sciascia and Radin, 2017) various medications (e.g., Abacavir, Zidovudine) (Sciascia and Radin, 2017; Apollonio et al., 2018; Darrow and Borisova, 2022), different diets (e.g., vegetarian) (Nucci et al., 2021), frostbite (Laurent and Vickers, 2009), hypothermia (Laurent and Vickers, 2009), carbon monoxide poisoning (Laurent and Vickers, 2009), hyperthermia (Laurent and Vickers, 2009), sunburn (Laurent and Vickers, 2009), insect bites (Laurent and Vickers, 2009), and women's health-related topic (e.g., discrimination) (Wang and Zhang, 2020). Therefore, the reviewed studies have been predominantly used in understanding health information utilization for various communicable and non-communicable diseases. Hence, future research can also focus on medications and health-related factors.

### 3.4. Purpose of using Wikipedia page views for health research

Studies utilized Wikipedia page views mainly to determine changes in the information usage of its pages (see Table 1). This curiosity toward Wikipedia page views as a metric of online health information usage may stem from its high use compared to other leading health websites, such as the World Health Organization and the National Institutes of Health (Heilman and West, 2015). The purpose and aims of the reviewed studies were categorized based on the analysis aim categorization of Nuti et al. (2014), which includes descriptive, causal reference, and surveillance. Causal inference studies aim to evaluate a hypothesized causal relationship with Wikipedia data, including statistical analysis. An example of a causal inference study is Gianfredi et al. (2023), which used Wikipedia data to assess the impact of a celebrity's announcement of having been diagnosed with pancreatic cancer on the trend of cancer-related research on the Internet. Descriptive studies describe the temporal or geographic trends of particular Wikipedia pages. An example of a descriptive study is Alibudbud (2023b), which described the worldwide utilization of online information for dementia. Finally, surveillance studies evaluated the use of Wikipedia page views to forecast or monitor real-world phenomena. An example of a surveillance study is O'Leary and Storey (2020), which shows a model for predicting the number of people who might become infected and die from COVID-19. Additionally, this review classified several studies as experimental studies, which are studies that measure the change in page views before and after editing Wikipedia pages. An example of an experimental study is Weiner et al. (2019), which enhanced Wikipedia health pages using high-quality research findings and tracked the persistence of those edits and the number of page views after the enhancement to assess the reach of this initiative.

**Table 1:** Purpose and recommended methodological considerations for studies utilizing Wikipedia page views for health research.

**Purpose of Wikipedia page views for health research**
1. To describe the changes in the utilization of online health information from Wikipedia at the country and global levels 2. To assess the impact of public events on public interest and information usage about health-related Wikipedia pages 3. To estimate and predict the incidence and prevalence of diseases 4. To predict data from other internet data sources 5. To evaluate the effectiveness of health education initiatives 6. To explore the evolution of a health topic
**Recommended checklist of methodological considerations for**
**studies utilizing Wikipedia page views for health research**
Specify: 1. Precise Wikipedia page or pages of study 2. Language of the Wikipedia page 3. Dates of data collection 4. Dates explored 5. Type of data (e.g., monthly or daily) 6. Whether page views were limited to Internet users 7. Whether web crawlers and redirects to the Wikipedia page were included

The most common aim of the reviewed studies was descriptive (*n* = 13, 44.83%), followed by causal inference (*n* = 6, 20.69%), surveillance (*n* = 6, 20.69%), and experimental (*n* = 4, 13.79%). Specifically, the present review found that data about Wikipedia page views were used to describe the changes and patterns in the utilization of online health information from Wikipedia at the country and global levels (Laurent and Vickers, 2009; Sciascia and Radin, 2017; Mahroum et al., 2018; Adams et al., 2020; Gozzi et al., 2020; Szmuda et al., 2020; Ciaffi et al., 2021; Nucci et al., 2021; Rutovic et al., 2021; Alibudbud and Cleofas, 2022; Mondia et al., 2022; Alibudbud, 2023b; Roe et al., 2023). In addition, it has also been utilized to assess the impact of public events, such as a celebrity's announcement of a disease, the death of a celebrity, media coverage of accidents and epilepsy, on public interest and information usage about different health-related Wikipedia pages (Brigo et al., 2015; Okumura et al., 2016; Naik et al., 2021; Gianfredi et al., 2023).

Wikipedia page views have also been used to compare and correlate with established epidemiological data and the burden of diseases with moderate to strong correlations (e.g., data from Istituto Superiore di Sanit) (Provenzano et al., 2019, 2021; Qiu et al., 2019; Gianfredi et al., 2021). In addition, it has been used in developing models that can be used to estimate and predict the incidence and prevalence of diseases such as influenza and coronavirus (O'Leary and Storey, 2020; De Toni et al., 2021). Likewise, it has been utilized to predict data from other internet data sources, such as the sentiment of tweets (Storey and O'Leary, 2022). Thus, the reviewed studies support that models using Wikipedia page views, similar to other sources of internet big data (e.g., Google Trends) (Alibudbud, 2023a), can be developed to forecast outbreaks of various health conditions.

Wikipedia has also been used to evaluate the effectiveness of institutional and school-based health education initiatives (e.g., Cochrane Russia Initiative) (Adams et al., 2020; Potapov et al., 2021). For example, the studies of Apollonio et al. (2018) and Weiner et al. (2019) showed that educational activities could be supplemented by having students edit Wikipedia pages and using their page views as activity indicators. Interestingly, the study by Wang and Zhang (2020) also used Wikipedia page views to explore the evolution of a particular health topic, Women's health.

Generally, the reviewed studies also showed that Wikipedia use for health-related information has changed over the years, which can persist in the future (Mahroum et al., 2018; Alibudbud and Cleofas, 2022; Darrow and Borisova, 2022; Alibudbud, 2023b). For instance, Alibudbud (2023b) predicts a decreasing utilization of Wikipedia for online dementia information, while Mahroum et al. (2018), Alibudbud and Cleofas (2022), and Darrow and Borisova (2022) showed an increasing trend of public utilization of online information from Wikipedia for substance use disorder, drugs, and chikungunya, respectively. Therefore, the reviewed studies show that previous notions of widespread use of Wikipedia for health information may vary depending on the health topic itself. The review also supports that future research can explore other health topics and areas to fully understand the utilization of Wikipedia for health information.

### 3.5. Data analysis of Wikipedia page views for health research

The reviewed studies were also categorized according to their data analysis using the data analysis categorization by Mavragani et al. (2018) of Google Trends data. This categorization includes visualization, seasonality, correlations, forecasting, modeling, and statistical tools. Studies considered under the visualization category include those with any form of visualization (e.g., figures and screenshots). Studies categorized under seasonality included those that explored the seasonality of their respective topic. Studies that have examined correlations are included in the correlations category. These correlations may be between Wikipedia data and other web-based sources (e.g., Google Trends). Forecasting studies include those that predicted future Wikipedia page views (e.g., ARIMA). Modeling studies employed some form of modeling using Wikipedia data (e.g., Structural Equation Modeling). For this review, the other statistical tools category includes studies, which utilized statistical tools aside from the ones in the previous categories (*t*-test and Wilcoxon sign rank test. The most common data analysis used by the reviewed studies was visualization (*n* = 20, 68.97%), followed by correlations (*n* = 8, 27.59%), modeling (*n* = 7, 24.14%), seasonality (*n* = 4, 13.79%), and forecasting (*n* = 3, 10.34%). About a quarter utilized other statistical tools (*n* = 7, 24.14%). Thus, similar to other utilized big data from the Internet used in health studies, such as Google Trends, future studies may further explore forecasting the use of Wikipedia for health information (Mavragani et al., 2018).

### 3.6. Recommended methodological considerations for future studies

Some of the reviewed studies may also be difficult to replicate due to some limitations in methodological information. These limitations in methodological information have also been observed in studies that use other big data on the Internet, such as Google Trends (Alibudbud, 2023a). For instance, some of the reviewed research, especially those studying a large amount of Wikipedia pages, did not mention or supplement their publication with the specific Wikipedia pages under study. Therefore, the details needed may not be enough to replicate their studies. In this regard, common methodological considerations that may enable replicability among the reviewed studies can be adapted in future studies using Wikipedia page views (Laurent and Vickers, 2009; Sciascia and Radin, 2017; Mahroum et al., 2018; Adams et al., 2020; Gozzi et al., 2020; Szmuda et al., 2020; Ciaffi et al., 2021; Nucci et al., 2021; Rutovic et al., 2021; Alibudbud and Cleofas, 2022; Mondia et al., 2022; Alibudbud, 2023b; Roe et al., 2023). As shown in Table 1, these methodological considerations can include specifying the precise Wikipedia page of study, the language of the Wikipedia page, the dates of data collection, the dates explored, the type of data (e.g., monthly or daily), and whether page views were limited to Internet users or web-crawlers and redirects to the Wikipedia page were included in the analysis.

### 3.7. Limitations of the present review

Although this review provided information on several uses of Wikipedia page views, its findings should be interpreted in light of its limitations. This review explored two of the world's largest research databases. Thus, future reviews can examine other databases that may contain studies about Wikipedia page views and health topics. Second, this review utilizes a limited number of keywords. Different keywords, such as “WikiTrends” and “Wiki”, can also be explored in future studies. Third, this review solely considered publications that included mentions of Wikipedia in their titles, abstracts, and keywords. As a result, studies that focused on Wikipedia but only mentioned it in their maintext, such as the article by Rustagi and Patel (2020), were not considered in the review. Therefore, the limited search scope may have overlooked other studies approaching the topic from different angles. Fourth, this review explored limited study characteristics. Future studies can explore other important study characteristics, such as the statistical analyses used in examining Wikipedia page views.

### 3.8. Conclusion

Wikipedia a widely read source of online health information. This review analyzed 29 studies utilizing Wikipedia page views for health research. Most of the reviewed studies have been published in recent years. Most reviewed studies also emanated from high-income countries. Alonside Wikipedia page views, these studies commonly incorporated data from Google, Twitter, YouTube, Reddit, and online news sources. The reviewed studies also predominantly explored non-communicable diseases and communicable diseases. Additionally, the utilization of Wikipedia page views in health research encompassed various purposes, including describing changes in online health information utilization, examining the impact of public events on public interest and information usage, estimating disease incidence and prevalence, predicting data from other internet sources, evaluating the effectiveness of health education initiatives, and exploring the evolution of health topics.

To address the limitations in replicating some of the reviewed studies, future studies can specify several methodological aspects, including the specific Wikipedia page(s) analyzed, the language of the Wikipedia pages examined, data collection dates, dates explored, type of data, and the inclusion web crawlers and redirects to the Wikipedia page(s). Because the pattern of Wikipedia usage varies depending on the health topic and the presence of public events, future research can look into other commonly read health topics. Future research can also develop models using Wikipedia page views that can be used to predict disease outbreaks and forecast the utilization of online health information. In addition, health education activities can be developed and explored using Wikipedia page views.

## Author contributions

RA had substantial contributions to the design, drafting, revision, acquisition, interpretation, and final approval of the data and work.
