# Supplementary material for: Wikipedia page views for health research: a review
Source: Front Big Data. 2023 Jul 4;6:1199060. doi: 10.3389/fdata.2023.1199060 (PMC10353851; doi:10.3389/fdata.2023.1199060)
Supplement: Supplementary file 1 [file Table_1.DOCX]

**Supplementary Files**

**Supplementary file 1. Review of studies utilizing Wikipedia page views for health research**

| **Author (Year)** | **Authors' Country of Origin** | **Wikipedia page** | **Purpose** | **Findings related to Wikipedia page views** | **Other utilized internet data** |
| --- | --- | --- | --- | --- | --- |
| Alibudbud (2023) | Philippines | Dementia | To describe the worldwide utilization of online information for dementia. | Online information about dementia increased and will further increase using Google in succeeding years. | Google Trends |
| Roe et al. (2023) | United States | Fencing Response | To quantify trends that demonstrate levels of public discussion and awareness of the fencing response over time. | Twitter mentions of the fencing response and Wikipedia page views increased overall from 2010 to 2019. | Twitter mentions |
| Gianfredi et al. (2023) | Italy | Fedez, Tumore del pancreas, and Tumore neuroendocrino | To assess the impact of a celebrity's announcement of having been diagnosed with pancreatic cancer on the volume of cancer-related research on the Internet. | Wikipedia Trends data indicated a moderate correlation for Tumore neuroendocrino and Tumore del pancreas. The search peaks for the GT and Wikipedia pages occur during the same time period. | Google Trends |
| Alibudbud and Cleofas (2022) | Philippines | Substance use disorder | To explore the worldwide information utilization for substance use disorder. | Wikipedia page views for substance use disorder-related information increased by almost 200% between 2015 and 2022. | Google Trends |
| Storey and O'Leary (2022) | United States | Coronavirus and COVID-19 | To analyze text of such user disclosures to study human communication during a pandemic. | Google and Wikipedia searches were predictive of sentiment in the tweets. | Google Trends, Twitter tweets |
| Darrow and Borisova (2022) | United States | All new drugs approved by the Food and Drug Administration (FDA) from 1982 to 2020 with associated Wikipedia pages | To determine the extent to which one prominent source of online information, Wikipedia, presented quantitative efficacy data about drugs. | Approximately 98% of 1201 drugs approved from 1982 to 2020 had Wikipedia pages. Most pages provided indirect indicia of efficacy. Wikipedia drug pages were associated with indicia of high impact, including a median of more than 23,000 annual page views. | None |
| Mondia et al. (2022) | Philippines | Various brain tumors (e.g., Glioblastoma, Meningioma, Glioma, Astrocytoma) | To describe the pattern of online search queries of keywords related to neoplasms of the central nervous system. | Search volumes from Google Trends and views of Wikipedia pages reflected comparable data in terms of known prevalence rankings of tumor subtypes. | Google Trends |
| Nucci et al. (2021) | Italy and Netherlands | Various diets (e.g., Mediterranean, vegetarian, ketogenic, Atkins) | To assess whether an analysis of Internet searches could provide information on the Internet users' behaviour/interest in diets. | The Mediterranean diet was the most frequently viewed, followed by the pescatarian diet. Significant seasonal differences were found for the Mediterranean, vegetarian, Atkins, Scarsdale, and zone diets and pescetarianism. | Google Trends |
| De Toni et al. (2021) | Italy and Spain | Various influenza related pages (e.g., Malattie infettive virali, Virusziekte, Virale Infektionskrankheit, Malattie infettive, Epidemie, Epidemie, Virus) | To show the feasibility of exploiting machine learning models and information about Wikipedia’s page views of a selected group of articles to obtain accurate estimates of influenza-like illnesses incidence in four European countries | A novel language-agnostic method based on two algorithms, Personalized PageRank and CycleRank, to automatically select the most relevant Wikipedia pages to be monitored without the need for expert supervision. | None |
| Naik et al. (2021) | Canada | Colorectal cancer | To quantify the impact of Chadwick Boseman's death on web-based search interest in colon cancer and determine whether there was an increase in interest in regions of the United States with a greater proportion of Black Americans. | There was a significant increase in web-based activity related to colon cancer following Chadwick Boseman's death, particularly in areas with a higher proportion of Black Americans. This | Google Trends |
| Gianfredi et al. (2021) | Italy | Influenza, Febbre and Tosse | To assess if the frequency of the Italian general public searches for influenza, using the Wikipedia web-page, are aligned with Istituto Superiore di Sanit (ISS) influenza cases. | Temporal correlation was observed between the bulletin of ISS and Wikipedia search trends | None |
| Provenzano et al. (2021) | Italy | Chikungunya, Dengue, Zika virus, and West Nile Virus | To evaluate the temporal correlation between Wikitrends and conventional surveillance data generated for Chikungunya, Dengue, Zika, and West Nile Virus infection reported by bulletin of Italian National Institute of Health (Istituto Superiore di Sanità in italian, ISS). | A correlation was observed between the bulletin of ISS and Wikipedia Wikitrends, the correlation was strong for Chikungunya and West Nile Virus, while the correlation was moderate for Dengue and Zika virus. | None |
| Rutovic et al. (2021) | Croatia, Argentina, Kyrgyzstan, and Italy | Stroke, multiple sclerosis, epilepsy, Alzheimer’s disease, Parkinson’s disease, and coronavirus. | To assess the total monthly number of views of the Wikipedia articles in English describing main neurological diseases in the period from January 2018 to July 2020 | A trend of a decrease in interest in neurological disease-related pages throughout years and especially during the burst of interest towards coronavirus. | None |
| Potapov et al. (2021) | Russian Federation | Intervention group: migraine treatment (13), diabetes mellitus (9), and pain and inflammation (14). Control group: articles on cardiovascular (11), gastrointestinal (14), and dermatological agents (11). | To evaluate the effectiveness of the Cochrane Russia Initiative to improve the articles of the Russian-language Wikipedia by including information from Cochrane Systematic Reviews (CSR) to ensure the accuracy and impartiality of their content as an information basis for the quality use of medicines by doctors and the public. | After “Cochrenization”, the number of Wikipedia article views increased in total/average article views for migraines, pain and inflammation, anddiabetes mellitus. Analysis of Cochrane reports showed an increase in the number of views of Cochrane systematic reviews summaries on the Cochrane.org website in general by 9 times and from Russian-speaking browsers by 11 times. | None |
| Ciaffi et al. (2021) | Italy | Lombalgia | To investigate the seasonal pattern of search volumes for back pain in Italy. | Cosinor analysis performed on views for the page "lombalgia" in Wikipedia confirmed a significant seasonality with apeak of interest in winter months and decrease in spring/summer. | Google Trends |
| Adams et al. (2020) | United Kingdom, Australia, Germany, and China | Wikipedia pages of direct relevance to up-to-date systematic reviews of the Cochrane Schizophrenia Group. | To investigate the effects of adding high-grade quantitative evidence of outcomes of treatments into relevant Wikipedia pages on further information-seeking behaviour by the use of routinely collected data. | For every person who sought and clicked the reference on the ‘intervention’ Wikipedia page to seek more information (the primary outcome), many more are likely to have been informed by the page alone. | John Wiley Ltd., Google Analytics |
| Gozzi et al. (2020) | United Kingdom, Italy, and United States | Wikipedia articles related to the COVID-19 (e.g., COVID-19 vaccine) | To characterize the media coverage and collective internet response to the COVID-19 pandemic in four countries: Italy, the United Kingdom, the United States, and Canada. | collective attention was mainly driven by media coverage rather than epidemic progression, rapidly became saturated, and decreased despite media coverage and COVID-19 incidence remaining high. | Online news, Youtube, reddit |
| Wang and Zhang (2020) | China and United States | Women’s health topic (e.g., discrimination) | To explore the evolutions of the women’s health topic on Wikipedia. | Women-health-related entries increased from 2010 to 2017, and nearly half of them were related to the supports and protection of women’s health. Total number of page views of the investigated items increased from 2011 to 2013, but it decreased from 2013 to 2017. | Wikipedia creation time, page edits data, and text of historical versions |
| O'Leary and Storey (2020) | United States | COVID-19 related pages | To develop an indicator model of the number of people in the USA who will become infected and die from the coronavirus. | Provided an indicator model of the number of people in the USA who will become infected and die from the coronavirus. | Google searches, Twitter tweets |
| Szmuda et al. (2020) | Poland and United Kingdom | Sciatica | To assess the online health interest in sciatica and lower back pain. | The Wikipedia page about sciatica has ranked high in all four categories it was included in, thus demonstrating that sciatica is a significant concern for the public in the years 2015-2019. Wikipedia pages about sciatica and low back pain had a respectively 28% and 90% increase in views from July 2015 to March 2019. | Google trends, pubmed data |
| Qiu et al. (2019) | United States | 1633 diseases (e.g., Viral Hepatitis) | To determine whether Internet usage and social media data correlated with the disease burden, measured by prevalence and treatment cost. | Wikipedia and Twitter data strongly correlated with the burdens of 15 and 7 diseases. | Google searches, Twitter tweets |
| Provenzano et al. (2019) | Italy | Morbillo, Vaccinazione del Morbillo, Vaccinazione MPR and Macchie di Koplik | To evaluate the temporal correlation between Wikitrends and conventional surveillance data generated for measles infection reported by bulletin of Istituto Superiore di Sanit (ISS). | temporal correlation was observed between the bulletin of ISS and Wikipedia search trends. The strongest correlation was at a lag of 0 for Measles, Measles Vaccination, MPR Vaccination and Koplik's spots. Regarding the database with weekly data, both moderate and strong correlation was observed. | None |
| Weiner et al. (2019) | United States | Various Wikipedia health pages | To enhance Wikipedia health pages using high-quality, current research findings and track the persistence of those edits and number of page views after the changes to assess the reach of this initiative. | Of the 24 edits, 22 persisted at the end of the observation period (from time of entry to May 31, 2018) and received nearly 8 million page views. Each health page had an average of 354,724 page views. | None |
| Apollonio et al. (2018) | United States, United Kingdom, Australia, and Canada | Various medicines (e.g., Abacavir, Valganciclovir, Zidovudine) | To expand on this traditional approach by having students improve actual medicines information pages posted on Wikipedia. | Engaging pharmacy students in a Wikipedia editing assignment is a feasible alternative to writing drug monographs as a classroom assignment. | None |
| Mahroum et al. (2018) | Israel and Italy | Chikungunya | To assess the Chikungunya-related digital behavior and the interplay between epidemiological figures and novel data streams traffic. | Between July 2015 and August 2017, there was a daily average of 69 pageviews. However, between September and October 2017 there was a very large burst of page traffic, resulting in an average of 3,862 daily active visits. | Google Trends, Google News and Twitter traffic, Wikipedia edits, and PubMed articles |
| Sciascia and Radin (2017) | Italy | Systemic Lupus Erythematosus (SLE) and belimumab | To investigate trends of Internet search volumes linked to SLE, on-going clinical trials and research developments associated to the disease, using Big Data monitoring and data mining. | By comparing the search volumes analysing the Wikipedia page views of both SLE and belimumab, a close peak trend, reflecting the knowledge translation after the approval of belimumab for the treatment of SLE. | Google Trends, scientific search tools (SCOPUS, Medline/ Pubmed/ ClinicalTrails.gov) |
| Okumura et al. (2016) | Japan | Epilepsy | To evaluate changes in the attitudes of nonmedical university students toward epilepsy in 2015, the present study compared the results of questionnaire surveys from four different time periods. | Analyses of information-seeking behavior on the Internet showed that the increase in Google search volume and Wikipedia page views was much less in 2015 than in 2011 and 2012. | Google Trends |
| Brigo et al. (2015) | Italy, Nigeria, Austria, and Netherlands | Alzheimer's disease; Amyotrophic lateral sclerosis; Dementia; Epilepsy; Epileptic seizure; Migraine; Multiple sclerosis; Parkinson's disease; Stroke; Traumatic brain injury | To evaluate Wikipedia page visits in relation to the most common neurological disorders by determining which factors are related to peaks in Wikipedia searches for these conditions. | Seven out of 10 neurological conditions showed relations in search volume peaks and news headlines. Six out of these seven peaks were related to news about famous people suffering from neurological disorders, especially those from showbusiness. | None |
| Laurent and Vickers (2009) | Belgium and United States | frostbite, hypothermia, carbon monoxide poisoning, common cold, pneumonia, bronchiolitis, norovirus, influenza, rhinovirus, seasonal affective disorder, hyperthermia, sunburn, hay fever, insect bites and stings, bee sting, Lyme disease, Rocky Mountain spotted fever, hemolytic-uremic syndrome, harvest mite and West Nile virus. | To determine the significance of the English Wikipedia as a source of online health information. | Wikipedia articles were viewed more often than MedlinePlus Topic. | Google, Google UK, Yahoo, and MSN, and page view statistics for selected MedlinePlus pages |
